# Liquiritoside Alleviated Pb Induced Stress in *Brassica rapa* subsp. *Parachinensis*: Modulations in Glucosinolate Content and Some Physiochemical Attributes

**DOI:** 10.3389/fpls.2021.722498

**Published:** 2021-08-26

**Authors:** Waheed Akram, Waheed Ullah Khan, Anis Ali Shah, Nasim Ahmad Yasin, Guihua Li

**Affiliations:** ^1^Guangdong Key Laboratory for New Technology Research of Vegetables/Vegetable Research Institute, Guangdong Academy of Agricultural Sciences, Guangzhou, China; ^2^Department of Environmental Science, The Islamia University of Bahawalpur, Bahawalpur, Pakistan; ^3^Department of Botany, University of Narowal, Narowal, Pakistan; ^4^RO-II Office, University of the Punjab, Lahore, Pakistan

**Keywords:** Chinese flowering cabbage, flavonoids, glucosinolates, liquiritoside, lead

## Abstract

Current research was conducted to explore the effects of liquiritoside on the growth and physiochemical features of Chinese flowering cabbage (*Brassica rapa* subsp. *parachinensis*) under lead (Pb) stress. Lead stressed *B. rapa* plants exhibited decreased growth parameters, chlorophyll, and carotenoid contents. Moreover, Pb toxicity escalated the synthesis of malondialdehyde (MDA), hydrogen peroxide (H_2_O_2_), flavonoids, phenolics, and proline in treated plants. Nevertheless, foliar application of liquiritoside mitigated Pb toxicity by decreasing oxidative stress by reducing cysteine, H_2_O_2,_ and MDA contents in applied plants. Liquiritoside significantly increased plant height, shoot fresh weight and dry weight, number of leaves, and marketable value of Chinese flowering cabbage plants exposed to Pb toxicity. This biotic elicitor also enhanced the proline, glutathione, total phenolics, and flavonoid contents in Chinese flowering cabbage plants exposed to Pb stress compared with the control. Additionally, total glucosinolate content, phytochelatins (PCs), and non-protein thiols were effectively increased in plants grown under Pb regimes compared with the control plants. Overall, foliar application of liquiritoside can markedly alleviate Pb stress by restricting Pb translocation in Chinese flowering cabbage.

## Introduction

Vegetables belonging to the Brassicaceae family show cherished health remunerations effects owing to the existence of biologically active and robust antioxidative ingredients. Chinese flowering cabbage (*Brassica rapa* subsp. *parachinensis*) is an annual cole crop belonging to the Brassicaceae family. The germination to harvest period of this vegetable is less than 60 days (Peng et al., 2015). Chinese flowering cabbage has valuable biological and nutritional properties (Aixia et al., 2015). Its above-ground parts, including leaves, stem, and inflorescence, can be cooked or consumed raw as salads. Its leaves contain adequate amounts of glucosinolates and polyphenolic compounds(Chen et al., 2008). This rich chemical composition and scientifically proven biological activity has made Chinese flowering cabbage a famous culinary plant (Chung et al., 2016).

Glucosinolates (GLs) are primarily found in plants of the genus *Brassica*, which include crops of economical and nutritional importance. GLs are rich in sulfur and anionic secondary metabolites (Johnson, 2002). GLs have been extensively studied for their protective effect against herbivory in plants and chemotherapeutic activity in humans (Barba et al., 2016). The consumption of vegetables containing glucosinolates may confer protection against cancer in humans (Johnson, 2002). The hydrolytic breakdown products of glucosinolates have beneficial effects on human health, including cytotoxic and apoptotic effects in damaged cells, and reducing risks of degenerative diseases (Barba et al., 2021).

Lead contamination in soil has rapidly increased during the last decades (Sidhu et al., 2016). Metal pollution severely affects the growth and development of metal-affected plants (Borges et al., 2019). Pesticides, fertilizers, and automobile fuel are the major sources of Pb contamination. This non-essential toxic metal impedes appropriate plant nutrition (Lamhamdi et al., 2013). The edible parts of plants may uptake higher levels of metal contaminant from polluted soils and hence become a health risk for human beings and livestock consumers (Baghaie and Fereydoni, 2019). Metal toxicity enhances oxidative injury by intensifying the synthesis of reactive oxygen species (ROS) (Malar et al., 2014). Metal stressed plants increase the antioxidative system to detoxify ROS and maintain their ionic homeostasis (Fayez et al., 2014). Lead stress also affects photosynthesis, transpiration, and ionic homeostasis in stressed plants (Devi et al., 2013). As well, the plants subjected to Pb toxicity demonstrate a higher level of lipid peroxidation and ROS (Tauqeer et al., 2016). Phenolics are secondary metabolites deposited in plants facing stress and play a defensive role against a higher level of ROS synthesized in these plants (Fayez et al., 2017).

Lethal effects of synthetics pharmaceuticals have augmented the discovery and large-scale production of natural bioactive molecules. But, the resources of natural bioactive compounds are inadequate for various reasons. Conversely, the consumer entreaty for these compounds is growing gradually. Hence, the application of novel approaches to fulfill the current growing demand for natural bioactive compounds is of immense relevance. The use of conventional approaches to accelerate natural biosynthetic pathways in plants is shown to produce high levels of bio-active compounds, without the need of genetic engineering applications (Trejo-Téllez et al., 2019). Further, advances in technology have augmented the discovery of new biotic elicitors capable of increasing the production of secondary metabolites in plants.

*Glycyrrhiza uralensis* Fisch (Fabaceae), commonly known as licorice, is a traditional plant recognized through the ages for its multiple health benefits and medicinal uses (Kondo et al., 2007; Tanemoto et al., 2015). The roots of this plant are used to treat influenza, coughs, and liver damage in traditional medicinal formulations (Kondo et al., 2007). Liquiritoside also known as liquiritin, is the biologically active component of licorice. This study was designed to investigate the influence of liquiritoside as a foliar spray to increase plant growth and concentration of specific biologically active substances such as proline, glucosinolates, phenolics, and flavonoids in Chinese flowering cabbage plants. In addition, Pb affects the growth of crop plants (Figlioli et al., 2019). However, there is a dearth of research work demonstrating the effect of Pb stress on *B. rapa* subsp. *parachinensis.* According to our information, the effect of Pb toxicity and the ameliorative role of liquiritoside in the mitigation of subsequent stress has never been studied before.

In the course of our study, foliar application of liquiritoside showed positive effects on the growth of Chinese flowering cabbage plants (data yet not published). Additionally, we observed that liquiritoside mitigated abiotic stress, improved biosynthesis of, glucosinolates besides, flavonoids and phenolics in treated plants (Akram et al., 2020). Yet, the application of exogenously applied liquiritoside in the mitigation of plant stress has never been inspected. Therefore, it was hypothesized that liquiritoside might also modulate the antioxidative system of plants to alleviate Pb toxicity. Henceforth, the fundamental purpose of the present study was to elucidate the impact of liquiritoside spray on the physiology and growth of the plants under Pb stress. The results of current research will help to identify the differences in physiochemical process in the liquiritoside applied Pb stressed plants, which will perhaps the valuable crop producers planning to use liquiritoside plants growing in Pb contaminated soils.

## Materials and Methods

### Plant Material

A preliminary study was performed to optimize the dose of liquiritoside. Plants of Chinese flowering cabbage (*Brassica rapa* subsp. *parachinensis*) were raised in plastic pots (12-inch) containing sterilized commercial potting mix. The greenhouse experiment conducted in the present study entailed growing plants in pots containing sterilized Tref Jiffy (United States) media, which were placed in greenhouse at 20/25 ± 3°C (night/day) under a 16-h photoperiod. Commercial-grade liquiritoside of 99% purity was obtained from Riotto Botanicals, Shaanxi, China. Foliar formulations of the elicitor were prepared at different concentrations including 0, 0.15, 0.25, 0.50, 0.75 and 1 g/L. Control plants were sprayed with distilled water. The application was performed at the trifoliate stage. The relative growth rate (RGR) was calculated over 5 d time spans, after 1 week of elicitor application using the formula:

$$R\,G\,R=\frac{I\,n\,W\,2-I\,n\,W\,1}{\mathrm{t2}-\mathrm{t1}}$$

Where W1 = initial shoot dry weight, W2 = final shoot dry weight, t2-t1 = growth period.

### Greenhouse Experiment

**FIGURE 1:**
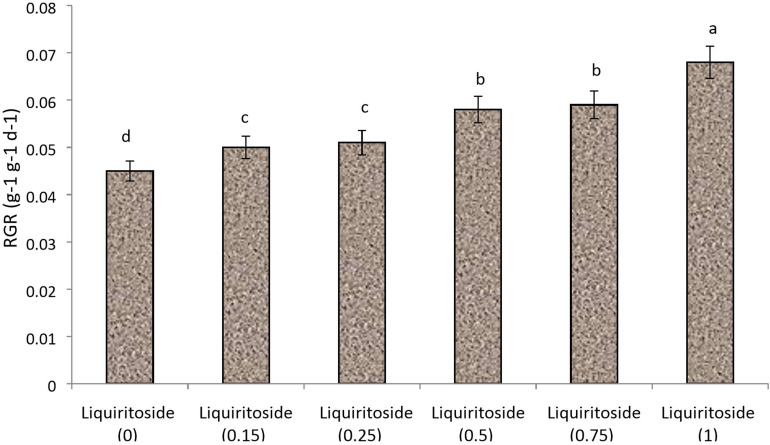
Effect of various doses of liquiritoside (g/L) on the relative growth rate (RGR) of Chinese flowering cabbage. Mean values of two independent experiments are presented. Vertical bars show standard error between different replicates of the same treatment. Data marked by different letters are significantly different at *p* < 0.05.

Based on RGR, an experimental set up was designed (Figure 1) under the same greenhouse conditions. Details of treatments are provided in Table 1. Seeds of Chinese flowering cabbage were sown in sterilized pots. An equal amount of water was supplied along with the recommended dose of fertilizers during the trial. The trial was carried out in a RCBD design containing 5 technical replicates of each treatment. Two independent greenhouse trials were conducted. No less than 10 plants were included in each treatment. Pb was added in the potting mix (75 mg/kg of potting mix) for artificial establishment of heavy metal stress according to the experiment design.

**TABLE 1:** Effect of liquiritoside on growth attributes of Chinese flowering cabbage plants under lead (Pb) stress.

Treatments	Plant Height	Number of leaves	Root FW	Shoot FW	Root DW	Shoot DW	Marketable value
	
	cm	–	g	g	g	g	(%age)
C	26.32 ± 1.78b	8.25 ± 0.35bc	15.5 ± 0.83b	81.29 ± 6.15b	6.56 ± 0.23b	54.75 ± 4.03bc	91.5 ± 5.41ab
Liquiritoside (0.25 g/L)	31.7 ± 2.19ab	9.57 ± 0.42ab	17.8 ± 0.92ab	87 ± 7.37ab	7.87 ± 0.24ab	58.08 ± 3.52b	92.4 ± 6.83a
Liquiritoside (0.50 g/L)	33.57 ± 2.38ab	10.38 ± 0.57ab	20 ± 1.25ab	95 ± 8.24ab	8.52 ± 0.27ab	61.31 ± 3.72ab	92.8 ± 6.91a
Liquiritoside (1 g/L)	35.62 ± 2.81a	12.43 ± 0.65a	22 ± 1.09a	98 ± 7.96a	8.93 ± 0.36a	68.26 ± 4.21a	93.3 ± 5.82a
Pb	18.3 ± 0.85c	6.38 ± 0.21c	9.37 ± 0.58c	64 ± 4.19c	4.15 ± 0.16c	31.17 ± 1.73d	60.4 ± 4.29c
Pb+ liquiritoside (0.25 g/L)	20.6 ± 0.93c	7.29 ± 0.26bc	12.98 ± 0.89bc	71 ± 5.73bc	6.31 ± 0.21bc	35.18 ± 2.87cd	67.8 ± 3.69bc
Pb+liquiritoside (0.50 g/L)	23.58 ± 1.15bc	8.26 ± 0.28bc	14.74 ± 0.76bc	76 ± 4.82bc	7.18 ± 0.29b	38.14 ± 2.43cd	72 ± 5.24b
Pb+liquiritoside (1 g/L)	25.16 ± 1.27b	8.91 ± 0.32b	15.63 ± 1.02b	82 ± 5.72b	8.43 ± 0.32ab	40.37 ± 2.84c	74 ± 4.82b

**Values presented are mean ± standard error of two independent experiments. Data marked by the different letters in the same column are significantly different at p < 0.05, C = Control, Pb = Lead (75 mg/kg soil).**

The elicitor was applied as a foliar application starting at the four-leaf stage and repeated on a fortnight basis. Plant growth parameters such as root/shoot length, root/shoot biomass, and the number of leaves were evaluated after 2 months of first foliar spray. The marketable value of plants was calculated using the following formula:

Marketablevalue(%)=100-(100×Percentageof injuredordiseasedplants/Percentageofhealthyplants).

A minimum of 20 plants was harvested from each treatment and was used for morphological and metabolomic analysis. Plant samples intended for metabolomics analysis were frozen in liquid nitrogen and kept at –80°C till examined.

### Assessment of Total Phenolic, and Flavonoids and Photosynthetic Pigments

Chlorophyll and carotenoids content was determined by using a spectrophotometer according to Metzner et al. (1965). The total phenolic contents were analyzed by employing the standard Folin-Ciocalteau method (Pavel et al., 2006) and expressed as milligrams gallic acid equivalent per gram of dry weight tissue. The total flavonoid content was estimated using the aluminum chloride colorimetric method of Chang et al. (2002).

### Analysis of the Nutritional Values of Leaves

Leaf samples for nutritional analysis were prepared as described by Mahmoud et al. (2019). Leaf samples were washed, stretched on paper towels, and air-dried for 60 min at room temperature. Thereafter, leaf samples were oven-dried at 70°C to ensure persistent weight. These dried samples were ground in a stainless-steel grinder and the following nutritional analyses were performed.

### Estimation of H_2_O_2_ and MDA

The amount of H_2_O_2_ content was estimated with the help of a spectrophotometer as described by Jana and Choudhuri (1981). The MDA level, a product of lipid peroxidation was quantified according to Heath and Packer (1968), with slight alterations as suggested by Zhang and Kirkham (1994).

### Assessment of Antioxidative Enzymes

The enzymatic activities of POD, SOD, and CAT were analyzed as described by Gao et al. (2005).

### Estimation of Phytochelatins (PCs), Non-protein Thiols (NPT), Cysteine, and Glutathione (GSH)

The cysteine content in the plant sample was measured according to the methodology of Gaitonde (1967). The amount of non-protein thiols content was estimated by employing the technique of Ellmann (1959). The quantity of sulfur-assimilating compounds was analyzed by adopting the technique of Nagalakshmi and Prasad (2001).

The amount of phytochelatins in treated plants was assessed according to Bhargava et al. (2005) by eliminating the quantity of GSH from the amount of NPTs as follows:

PCs=NPTs⁢-⁢GSH

### Quantification of Proline Content

The technique of Bates et al. (1973) was used for the estimation of proline content.

### Estimation of Lead

The quantity of Pb from digested plant samples was evaluated according to Khan et al. (2017) by using Atomic Absorption Spectrophotometer.

### Quantification of Glucosinolate Content

We used our recently devised method (Akram et al., 2020) for the identification and quantification of different types of glucosinolates from the leaves of Chinese flowering cabbage plants. Leaves from 10 plants were taken from each treatment and pooled together. Analysis was performed on an API 4000 QTrap mass spectrometer equipped with a TurboIonSpray probe (AB Sciex; Foster City, CA, United States) connected to a Shimadzu UFLC (Shimadzu, Kyoto, Japan). The mass spectrometer worked with triple quadrupole analyzer in the Multiple Reaction Monitoring (MRM) mode. Sinigrin does not exist in *B. rapa* and *B. napus* (Rangkadilok et al., 2002). In this study, it was used as an internal standard for quantitative analysis of glucosinolates (Jacobo-Velázquez et al., 2011).

### Statistical Analysis

The data obtained were analyzed by taking variance with the help of DSAASTAT software. The Duncan’s new multiple range test was employed for evaluation of the significant difference between means of all treatments. The study trials having three replicates were repeated twice, and values of means acquired are exhibited.

## Results

### Influence of Liquiritoside on Plant Height, Number of Leaves, Root Biomass, Shoot Biomass, and Marketable Value of Chinese Flowering Cabbage Subjected to Pb Stress

In the absence of Pb, the liquiritoside (1 g/L) treatment significantly increased the plant height, the number of leaves, marketable value, dry weight of the roots and shoots by 26, 34, 3, 27, and 20%, respectively, as compared to untreated control (Table 1). Liquiritoside (0.5 g/L) treatment had no significant effect on plant height, the number of leaves, marketable value, shoot, and root biomass after and before Pb exposure. In the presence of Pb, liquiritoside (1 g/L) application remarkably increased plant length, number of leaves, marketable value, the dry weight of the shoots and roots of Chinese flowering cabbage in comparison with Pb control (Table 1).

### Effects of Liquiritoside on Chlorophyll and Carotenoid Contents, Flavonoids, and Total Phenolics in Chinese Flowering Cabbage Under Pb Stress

As shown in Table 2, the contents of chlorophyll *a*, chlorophyll *b*, and carotenoids in plant tissues of the control treatment were 1.17, 0.47, and 2.78 mg g^–1^, respectively. The Pb treatment decreased the chlorophyll *a* and *b* and carotenoids contents in Chinese flowering cabbage by 27, 28, and 24%, respectively, than that of the untreated control. On the other hand, liquiritoside (1 g/L) supplementation significantly improved the chlorophyll *a* and *b* and carotenoids levels of plants exposed to Pb stress compared with relevant control.

**TABLE 2:** Effect of liquiritoside on chlorophyll, carotenoids, flavonoids, and phenolics levels of Chinese flowering cabbage plants under lead (Pb) stress.

Treatments	Chlorophyll *a*	Chlorophyll *b*	Total Chl.	Carotenoids	Flavonoids	Total phenolics
	
	mg g^–1^ FW	mg g^–1^ FW	mg g^–1^ FW	mg g^–1^ FW	mg of quercetin g^–1^	mg of GAE g^–1^
C	1.17 ± 0.052bc	0.47 ± 0.021b	1.64 ± 0.058bc	2.78 ± 0.13c	5.54 ± 0.25d	138 ± 6.17e
Liquiritoside (0.25 g/L)	1.32 ± 0.038b	0.53 ± 0.023ab	1.85 ± 0.072b	3.21 ± 0.18bc	6.62 ± 0.31c	145 ± 6.32de
Liquiritoside (0.50 g/L)	1.54 ± 0.073ab	0.61 ± 0.025ab	2.15 ± 0.085ab	3.48 ± 0.21b	7.34 ± 0.34bc	154 ± 7.24de
Liquiritoside (1 g/L)	1.65 ± 0.081a	0.64 ± 0.028a	2.29 ± 0.089a	4.36 ± 0.25a	7.86 ± 0.38bc	164 ± 7.4d
Pb	0.86 ± 0.042d	0.34 ± 0.016c	1.20 ± 0.036d	2.12 ± 0.09d	8.13 ± 0.42b	201 ± 9.6c
Pb+ liquiritoside (0.25 g/L)	0.95 ± 0.046cd	0.39 ± 0.015bc	1.34 ± 0.038cd	2.56 ± 0.13cd	9.24 ± 0.47ab	228 ± 12b
Pb+liquiritoside (0.50 g/L)	0.98 ± 0.056cd	0.42 ± 0.018bc	1.40 ± 0.041cd	2.76 ± 0.15c	9.06 ± 0.52ab	253 ± 13ab
Pb+liquiritoside (1 g/L)	1.12 ± 0.062c	0.45 ± 0.023b	1.57 ± 0.045c	2.94 ± 0.16bc	9.75 ± 0.58a	269 ± 15a

**Values presented are mean ± standard error of two independent experiments. Data marked by the different letters in the same column are significantly different at p < 0.05, C = Control, Pb = Lead (75 mg/kg soil).**

The results showed that Pb toxicity markedly augmented the level of both flavonoids, and phenolics by 32 and 38%, respectively, in Chinese flowering cabbage plants as compared to untreated controls. However, liquiritoside supplementation further boosted the magnitude of both flavonoids, and phenolics in the subject plants under Pb toxic and nontoxic conditions (Table 2). The Pb+liquiritoside (1 g/L) treatment accelerated the quantities of both flavonoids, and phenolics by 17 and 31%, respectively, in Chinese flowering cabbage with respect to only Pb treated plants.

### Impact of Added Liquiritoside on Antioxidant Enzyme Activities in Chinese Flowering Cabbage Under Pb Treatment

**FIGURE 2:**
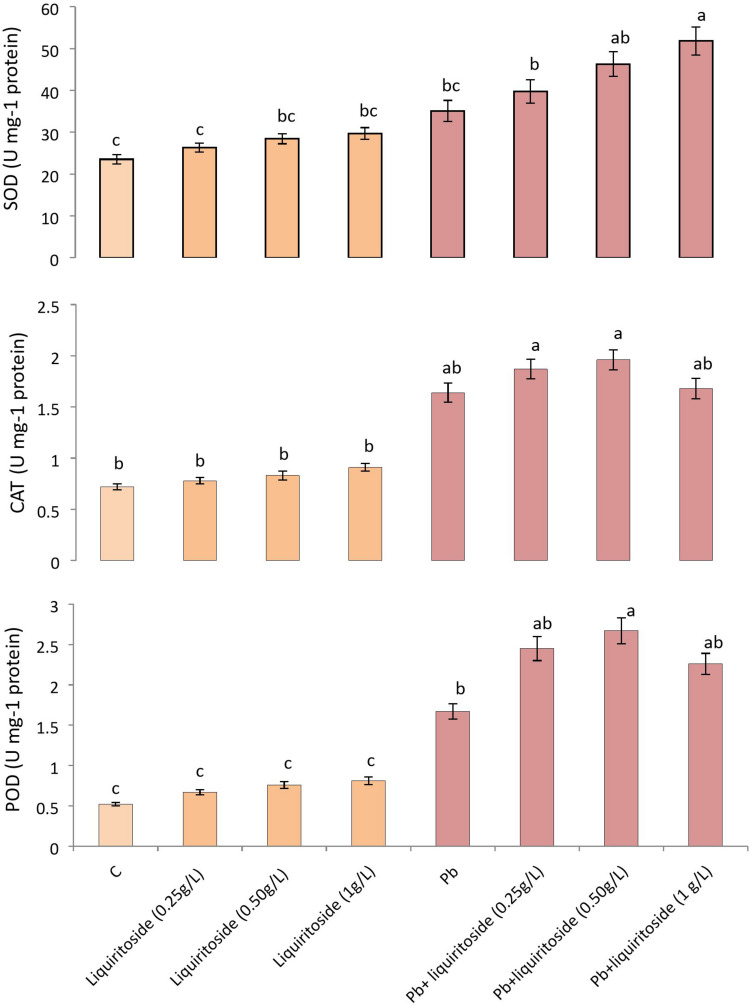
Effect of liquiritoside on the magnitudes of antioxidant enzymes such as SOD, POD, and CAT in Chinese flowering cabbage plants under lead stress. Mean values of two independent experiments are presented. Vertical bars show standard error between different replicates of the same treatment. Data marked by different letters are significantly different at *p* < 0.05. Pb, lead; C, Control.

Lead stress attenuated the activities of antioxidant enzymes like SOD, CAT, and POD in Chinese flowering cabbage plants when compared with untreated control. While supplementation of liquiritoside at different concentrations further modulated the level of these antioxidant enzymes (SOD, CAT, and POD) in plants subjected to Pb stressed and non-stressed regimes. The Pb+liquiritoside (0.5 g/L) exhibited remarkable increment in the level of SOD, CAT, and POD by 24, 16, and 37, respectively, in Chinese flowering cabbage plants in contrast with only Pb treated ones (Figure 2).

### Implications of Liquiritoside in the Modulation of GSH, PCs, Cysteine, and NPT Contents in Chinese Flowering Cabbage Exposed to Pb Toxicity

In the absence of Pb treatments, no obvious changes were observed in the GSH contents of plants with increased liquiritoside application, but the GSH contents were meaningfully different in plants raised under various liquiritoside treatments during Pb stressed conditions (Table 3). The pre-incubation of liquiritoside at the dose of 0.25, 0.50, and 1 g/L increased glutathione contents by 11, 14, and 18%, respectively, in Chinese cabbage plants raised under Pb regimes with respect to relevant controls. In the presence of Pb toxicity, sufficient and excessive liquiritoside treatment obviously increased the PCs and NPT contents in Chinese flowering cabbage plants compared with those plants in the liquiritoside deficiency treatment group. The, Pb+liquiritoside (1 g/L) treatment significantly enhanced the level of PCs and NPT in subjected plants by 14 and 17%, respectively, with respect to only Pb treated ones (Table 3). These findings signposted that the biosynthesis of thiol metabolic compounds (NPT, GSH, and PCs) enhanced with increased liquiritoside application in Chinese flowering cabbage to alleviate Pb stress. During the current study, liquiritoside (1 g/L) treatment noticeably attenuated the cysteine contents of Chinese flowering cabbage under nontoxic and toxic regimes (Table 3).

**TABLE 3:** Effect of liquiritoside on the levels of phytochelatins (PCs), cysteine, non-protein thiols (NPT), glucosinolates, glutathione (GSH) and proline in Chinese flowering cabbage plants under lead (Pb) stress.

Treatments	PCs	Cysteine	NPT	Total glucosinolates	GSH	Proline
	
	nmol-SH mg^–1^	nmol-SH mg^–1^	nmol-SH mg^–1^	μmol g^–1^	μmol g^–1^	μmol g^–1^
	protein	protein	protein	DW	FW	FW
C	294.51 ± 14.16cd	115.72 ± 6.18c	490.52 ± 21d	16.45 ± 0.73b	195.38 ± 9.34cd	18.65 ± 1.45e
Liquiritoside (0.25 g/L)	301.32 ± 16.27c	112.78 ± 6.41cd	483.67 ± 19de	18.19 ± 0.87ab	182.61 ± 8.71d	20.31 ± 1.53e
Liquiritoside (0.50 g/L)	289.42 ± 15.28cd	106.83 ± 5.23cd	476.76 ± 23de	21.62 ± 1.28ab	187.23 ± 9.28d	24.86 ± 2.54de
Liquiritoside (1 g/L)	272.74 ± 13.39d	94.91 ± 4.57d	465.81 ± 24e	25.24 ± 1.36a	193.92 ± 10.26cd	31.92 ± 2.65d
Pb	304.09 ± 17.53c	151.64 ± 7.83a	543.67 ± 25c	12.25 ± 0.71c	241.72 ± 12.75c	43.42 ± 3.83c
Pb+ liquiritoside (0.25g/L)	328.74 ± 18.79b	117.87 ± 8.75c	597.45 ± 28b	16.17 ± 1.23b	269.63 ± 13.68b	58.27 ± 2.71b
Pb+liquiritoside (0.50 g/L)	335.28 ± 21.94ab	128.96 ± 7.87bc	613.67 ± 32ab	15.94 ± 0.78b	278.17 ± 14.56ab	64.16 ± 3.62ab
Pb+liquiritoside (1 g/L)	351.83 ± 19.37a	134.68 ± 8.13b	642.26 ± 34a	17.39 ± 0.98ab	291.29 ± 16.46a	71.35 ± 4.39a

**Values presented are mean ± standard error of two independent experiments. Data marked by the different letters in the same column are significantly different at p < 0.05, C = Control, Pb = Lead (75 mg/kg soil).**

### Role of Liquiritoside on Total Glucosinolates and Proline Contents of Chinese Flowering Cabbage

Lead toxicity reduced the total glucosinolates by 26% in Chinese flowering cabbage plants than that of the untreated control. However, liquiritoside supplementation augmented the level of total glucosinolates during stressed and non-stressed conditions. The application of liquiritoside at the concentration of 0.5 and 1 g/L increased the value of total glucosinolates by 23 and 29%, respectively, in plants grown under metal regimes with respect to Pb control (Table 3). Present results depicted that lead stress remarkably declined the level of glucosinolate contents including Progoitrin, Glucoalyssin, Gluconapin, Glucobrassicin, Neoglucobrassin, 4- Hydroxyglucobrassicin and, 4-Methoxyglucobrassicin in Chinese flowering cabbage plants with respect to analogous untreated controls, respectively. Nevertheless, liquiritoside application enhanced the values of these glucosinolate contents in Chinese flowering cabbage plants under stressed and non-stressed regimes (Table 4).

**TABLE 4:** Effect of liquiritoside on glucosinolate contents in Chinese flowering cabbage plants under lead (Pb) stress.

Treatments	Progoitrin	Glucoalyssin	Gluconapin	Glucobrassicin	Neoglucobrassin	4– Hydroxyglucobrassicin	4–Methoxyglucobrassicin
	
	μmol g^–1^	μmol g^–1^	μmol g^–1^	μmol g^–1^	μmol g^–1^	μmol g^–1^	μmol g^–1^
	DW Sinigrin eqv.	DW Sinigrin eqv.	DW Sinigrin eqv.	DW Sinigrin eqv.	DW Sinigrin eqv.	DW Sinigrin eqv.	DW Sinigrin eqv.
C	0.58 ± 0.031ab	0.143 ± 0.0081ab	1.62 ± 0.051b	0.128 ± 0.0057ab	4.38 ± 0.24b	0.072 ± 0.0038e	2.46 ± 0.12b
Liquiritoside (0.25 g/L)	0.62 ± 0.032ab	0.152 ± 0.0086ab	1.67 ± 0.069ab	0.129 ± 0.0059ab	5.61 ± 0.31ab	0.078 ± 0.0043e	2.92 ± 0.14ab
Liquiritoside (0.50 g/L)	0.64 ± 0.035ab	0.163 ± 0.0092ab	1.76 ± 0.072ab	0.132 ± 0.62ab	6.03 ± 0.42ab	0.086 ± 0.0049e	3.37 ± 0.17ab
Liquiritoside (1 g/L)	0.67 ± 0.039a	0.174 ± 0.0091a	1.81 ± 0.084a	0.134 ± 0.0065a	6.34 ± 0.46a	0.092 ± 0.0052d	3.75 ± 0.19a
Pb	0.32 ± 0.021c	0.086 ± 0.0053b	0.97 ± 0.035d	0.075 ± 0.0027b	2.84 ± 0.14d	0.042 ± 0.0024c	1.58 ± 0.095d
Pb+ liquiritoside (0.25 g/L)	0.35 ± 0.024bc	0.091 ± 0.0061b	1.045 ± 0.042cd	0.079 ± 0.0029b	3.13 ± 0.16cd	0.049 ± 0.0027b	1.69 ± 0.098cd
Pb+liquiritoside (0.50 g/L)	0.39 ± 0.025bc	0.095 ± 0.0064b	1.167 ± 0.043cd	0.086 ± 0.0032b	3.27 ± 0.19cd	0.056 ± 0.0032ab	1.74 ± 0.10cd
Pb+liquiritoside (1 g/L)	0.42 ± 0.027b	0.098 ± 0.0068b	1.26 ± 0.048c	0.091 ± 0.0037b	3.49 ± 0.21c	0.063 ± 0.0034a	1.82 ± 0.11c

**Values presented are mean ± standard error of two independent experiments. Data marked by the different letters in the same column are significantly different at p < 0.05, C = Control, Pb = Lead (75 mg/kg soil).**

During the present research, proline content was enhanced by 57% in Chinese flowering cabbage exposed to Pb stress when compared with untreated control. While, differential liquiritoside (0.25, 0.5, and 1 g/L) supplementation further increased the level of proline by 26, 32, and 39%, respectively, in the subject plants under contaminated regimes than that of Pb control treatment (Table 3).

### Effect of Liquiritoside on MDA and H_2_O_2_ Concentrations in Chinese Flowering Cabbage Under Pb Stress

**FIGURE 3:**
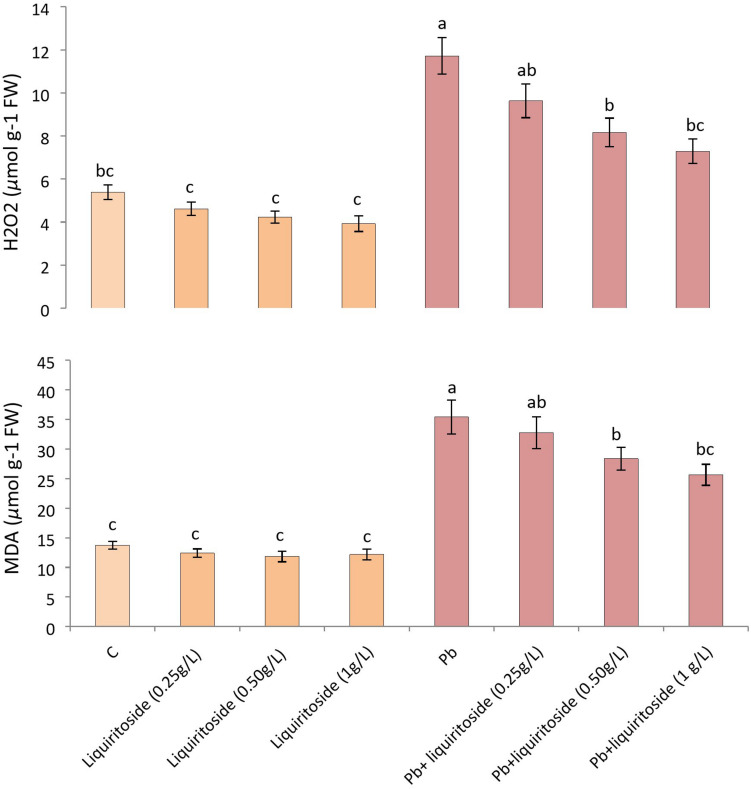
Effect of liquiritoside on the amounts of malondialdehyde (MDA) and hydrogen peroxide (H_2_O_2_) in Chinese flowering cabbage plants under lead stress. Mean values of two independent experiments are presented. Vertical bars show standard error between different replicates of the same treatment. Data marked by the different letters in the same column are significantly different at *p* < 0.05. Pb, lead; C, Control.

During the present investigation, Pb toxicity considerably augmented the contents of MDA and H_2_O_2_ in Chinese flowering cabbage with respect to untreated control. Nevertheless, excessive liquiritoside supplementation meaningfully declined the level of both MDA and H_2_O_2_ in plants during toxic and nontoxic circumstances. The Pb+ liquiritoside (1 g/L) treatment diminished the quantities of both H_2_O_2_ and MDA in Chinese flowering cabbage by 38 and 29%, respectively, as compared to concerned Pb treated groups (Figure 3).

### Role of Liquiritoside on Pb Uptake in Roots and Shoots of Chinese Flowering Cabbage

The findings of the current study depicted that root tissues showed more Pb uptake compared with shoot tissues of Chinese flowering cabbage (Table 5). The higher Pb accumulation was noticed in roots and shoots of plants grown under only Pb treatment. The liquiritoside supplementation exhibited a reduction of Pb uptake in the root and the shoot tissues of Chinese flowering cabbage as compared to only the Pb treatment group (Table 5). With the increase in liquiritoside doses (0.25, 0.5, and 1 g/L), a Pb uptake reduction was recorded in analyzed root and shoot tissues. The excessive dose liquiritoside (1 g/L) caused 28 and 43% decline of Pb uptake in root and shoot organs of Chinese flowering cabbage in contrast with non -supplemented stressed plants. The application of liquiritoside (1 g/L) significantly reduced the translocation factor and bio-concentration factor of Pb in subjected plants than that of non-supplemented ones. The plants grown under Pb regimes exhibited decreased value flowering cabbage plants in a dose dependent manner.

**TABLE 5:** Role of liquiritoside on Pb uptake in root and shoot tissues, bio-concentration factor (BCF), translocation factor (TF) and tolerance index (TI) of Chinese flowering cabbage plants under lead (Pb) stress.

Treatments	Root Pb uptake	Shoot Pb uptake	BCF	TF	TI
	
	mg kg^–1^	mg kg^–1^	–	–	%
C	0.86 ± 0.052c	0.52 ± 0.028c	0.55 ± 0.021c	0.60 ± 0.035a	–
Liquiritoside (0.25 g/L)	0.72 ± 0.045c	0.43 ± 0.021c	0.46 ± 0.024cd	0.59 ± 0.027a	1.21 ± 0.057ab
Liquiritoside (0.50 g/L)	0.58 ± 0.028c	0.31 ± 0.019c	0.35 ± 0.017cd	0.53 ± 0.024ab	1.27 ± 0.073ab
Liquiritoside (1 g/L)	0.47 ± 0.031b	0.23 ± 0.012c	0.28 ± 0.016c	0.48 ± 0.022b	1.35 ± 0.083a
Pb	41.09 ± 2.53a	25.64 ± 1.06a	0.87 ± 0.051a	0.62 ± 0.034a	0.69 ± 0.025c
Pb+ liquiritoside (0.25 g/L)	39.74 ± 2.62ab	18.87 ± 0.85ab	0.78 ± 0.042ab	0.47 ± 0.026bc	0.78 ± 0.038bc
Pb+liquiritoside (0.50 g/L)	36.28 ± 2.54ab	15.96 ± 0.79b	0.69 ± 0.034b	0.43 ± 0.021c	0.89 ± 0.056bc
Pb+liquiritoside (1 g/L)	29.83 ± 1.97b	14.68 ± 0.64b	0.59 ± 0.035bc	0.49 ± 0.025b	0.95 ± 0.047b

**Values presented are mean ± standard error of two independent experiments. Data marked by the different letters in the same column are significantly different at p < 0.05, C = Control, Pb = Lead (75 mg/kg soil).**

## Discussion

In the past few years, several studies have shown that exogenous elicitors can mediate plant growth and productivity (Bibi et al., 2016; Brockman and Brennan, 2017; Colla et al., 2017). To the best of our knowledge, the present study is the first to report the positive effects of liquiritoside on the growth and health-promoting elements of Chinese flowering cabbage grown under Pb stress. The application of genistein, a flavonoid improved growth and production of salted soya bean plants (Miransari and Smith, 2007). Likewise, coumarin, a phenolic compound enhanced the growth of wheat plants exposed to salt stress. Similarly, apigenin also enhanced biomass production and the growth of paddy plants under salt stress (Mekawy et al., 2018). Similarly, it was revealed that liquiritoside enhanced growth-related attributes and marketable value of Chinese flowering cabbage exposed to Pb regimes.

The increased growth rate of Chinese flowering cabbage plants observed under the influence of the foliar elicitor could be attributed to the capability of liquiritoside to modulate phytohormones, soluble sugars, amino acids, and mineral elements in applied plants. The liquiritoside may improve the yield and quality of Chinese flowering cabbage by affecting cellular metabolism. For example, it is known that sugars act as signaling molecules and improve plant growth and development (Smeekens et al., 2010). Amino acids provide improved stress tolerance in plants (Karabudak et al., 2014). Some organic acids present in plant extracts can chelate metal ions to stimulates root growth (Battacharyya et al., 2015). All these together could supply nutrition for cell growth, with a resulting increase in growth and vigor.

Chinese flowering cabbage plants under Pb stress exhibited a reduced level of photosynthetic pigments. Several other studies have revealed that Pb deteriorated chlorophyll structure and decreased chlorophyll synthesis by replacing Fe, Mg, and Cu (Akinci et al., 2010; Ashraf et al., 2016). Foliar application of liquiritoside positively affected total chlorophyll and carotenoids content in a dose-dependent manner (Table 2). The positive effect of liquiritoside on leaf pigment content could be attributed to the delay in leaf senescence or enhancement in leaf pigment biosynthesis (Fan et al., 2013; Jannin et al., 2013). These beneficial effects are possibly due to the effect of liquiritoside on phytohormones. The physiological parameter of leaf pigment content also acts as indicators of improved quality of Chinese flowering cabbage that can be obtained by the application of exogenous elicitors. Analogous to our results, it was observed that apigenin-treated plants showed increased biosynthesis of photosynthetic pigments which improved the growth of paddy plants subjected to salinity stress (Mekawy et al., 2018). Moreover, the cinnamic acid applied plants also exhibited an increased amount of photosynthetic pigments besides increased growth of maize plants exposed to salt toxicity (Araniti et al., 2018).

The enzymatic and non-enzymatic antioxidants enable plants to thrive under abiotic stress conditions (Usman et al., 2020). The antioxidative compounds perform the role of sacrificial agents through their activity on ROS, thus defending plant biomolecules. Rutin as an antioxidative flavonoid, scavenge ROS, and enhance the growth of leguminous plants (Ismail et al., 2016). Quercetin synthesized by rutin has ROS scavenging capability because it acts as a substrate of guaiacol peroxidase (Amako et al., 1994). The detoxification of ROS in quercitin applied plants mitigated salt-induced stress and improved the growth of plants. The antioxidant enzymes such as POD, CAT, and SOD consume phenolics, including cinnamate, ellagate and ferulate to alleviate plant stress (Abu Taleb et al., 2013; Singh et al., 2013). Similarly, our finding also exhibited obvious modulations in the activities of POD, CAT, and SOD for dilution of Pb toxicity in liquiritoside supplemented Chinese flowering cabbage plants.

Fayez et al. (2014) demonstrated that abiotic stress modulates physiochemical attributes of plants. Plants synthesize an elevated level of MDA and H_2_O_2_ under stress (Noctor et al., 2015). Lead stress enhanced the biosynthesis of ROS, leading to increased oxidative injury in plants (Hattab et al., 2016). Other studies also showed that Pb enhanced lipid peroxidation in plants causing oxidative injuries (Li et al., 2013). Plants engage the antioxidant system to mitigate metal-triggered oxidative stress (Shahid et al., 2014). Phenolics and flavonoids may reduce the biosynthesis of ROS, EL H_2_O_2_, and MDA to alleviate plant stress (Mekawy et al., 2018). The reduced level of ROS helps in the mitigation of oxidative injury (Hossain et al., 2019). The improved synthesis of phenolics and flavonoids as well as antioxidant enzymes detoxify ROS and mitigate stress in gallic acid, and rutin treated plants under stress. Pheomphun et al. (2019) observed that reduced H_2_O_2_, MDA, and enhanced activity of antioxidant enzymes in catechin supplemented plants for the alleviation of stress.

Higher proline has been observed in plants facing stress (Ahmad et al., 2018). However, exogenously applied quercetin and coumarin improve proline content, and LRWC in plants to mitigate stress (Saleh and Madany, 2015). The increased proline biosynthesis was attributed to reduced activity of proline dehydrogenase and increased activity of pyrroline-5-carboxylate synthase in coumarin supplemented plants (Pérez-Arellano et al., 2010; Szabados and Savoure, 2010). Hence, it is assumed that liquiritoside may mitigate metal stress in applied Chinese flowering cabbage plants in the same manner.

Phenolics scavenge ROS to reduce oxidative injury in plants (Soares et al., 2019). Our results showed that liquiritoside enhanced phenolic contents and triggered the activity of antioxidant enzymes. Phenolics detoxify ROS and metal toxicity by making metal complex in plants (Tolrà et al., 2009). The results of this study are in agreement with the findings of Ashraf et al. (2016), who observed that the foliar application of plant extracts increased the total phenolics and flavonoid content of *Raphanus sativus* plants. Baenas et al. (2014) showed that the nutritional quality of sprouts of brassica vegetables was improved by foliar application of biotic elicitors. Agati and Tattini (2010) reported that flavonoids decline in ROS levels in plants affected by abiotic stress. Similarly, other researchers have described the importance of GSH, flavonoids, and ascorbic acid in mitigation of plant stress through reducing ROS synthesis (Liang et al., 2018). The augmented synthesis of flavonoid alleviates drought stress in plants (Varela et al., 2016).

Current results showed that the levels of total GLS in Chinese flowering cabbage plants exposed to Pb toxicity were significantly increased under the influence of the foliar elicitor (1 g/L) (Table 3). Same types of increments of GLSs have been reported in Chinese flowering cabbage in previous studies (Bhandari et al., 2015; Liang et al., 2018). Metal stressed plants modulate the synthesis of thiol ligands, including phytochelatins (PCs), non-protein thiols, cysteine, and GSH for detoxification and chelation of metal (Kumar et al., 2016; Ahmad et al., 2020). Higher synthesis of thiols in root tissues compared to foliage of plants declines uptake and translocation of injurious metals from roots to shoots (Hasan et al., 2015). The thiol-containing groups of plants such as cysteine, NPT and PCs, have a higher affinity for metals and hence assist in homeostasis and detoxification of metals (Rabêlo et al., 2018). Similarly, cysteine, NPT, GSH, and PCs may have played their part reducing Pb translocation and subsequent detoxification in Chinese cabbage flowering plants.

Plant roots immediately come in contact with metals and hence exhibit relatively more metal content as compared to above-ground parts of plants. The increased demethylation and pectin level help in reduced translocation besides the fixation of metal within the root cell walls (Liu et al., 2019). Perhaps, this strategy reduced Pb translocation from root to shoot of the plants and should be explored in further studies (Bharwana et al., 2013).

## Conclusion

This study demonstrates that liquiritoside could be used as an effective plant growth bio-stimulant. Our findings indicate that the nutritional and medicinal contents in leaves of Chinese flowering cabbage plants can be augmented by foliar application of liquiritoside at a rate of 0.5 g/L. The supplementation of liquiritoside alleviated Pb stress of plants by improving growth/photosynthetic pigments, glucosinolates, antioxidants, and reducing MDA, H_2_O_2_, cysteine, and Pb uptake. Further studies are required to understand the mechanism underlying the crop’s growth effect, promoting biotic elicitor’s use in organic agriculture.

## Data Availability Statement

The original contributions presented in the study are included in the article/supplementary material, further inquiries can be directed to the corresponding author/s.

## Author Contributions

WA: suggest the idea and perform the experiments. WUK: carry out statistical analysis. NAY: writing manuscript. AS: data analysis and manuscript drafting. GL: research designing and supervision. All authors contributed to the article and approved the submitted version.

## Conflict of Interest

The authors declare that the research was conducted in the absence of any commercial or financial relationships that could be construed as a potential conflict of interest.

## Publisher’s Note

All claims expressed in this article are solely those of the authors and do not necessarily represent those of their affiliated organizations, or those of the publisher, the editors and the reviewers. Any product that may be evaluated in this article, or claim that may be made by its manufacturer, is not guaranteed or endorsed by the publisher.
